# Oral Contraceptive and Glioma Risk: A Prospective Cohort Study and Meta-Analysis

**DOI:** 10.3389/fpubh.2022.878233

**Published:** 2022-07-14

**Authors:** Chuan Shao, Hui Tang, Xiaoya Wang, Jiaquan He, Pan Wang, Nan Wu

**Affiliations:** ^1^Department of Neurosurgery, Chongqing General Hospital, Chongqing, China; ^2^Department of Neurosurgery, Nanchong Central Hospital, The Second Clinical Medical College, North Sichuan Medical College, Nanchong, China; ^3^Graduate Institute, Chongqing Medical University, Chongqing, China

**Keywords:** oral contraceptive, glioma, risk factors, cohort, meta-analysis

## Abstract

**Background:**

Epidemiological evidence that glioma has a slight male predominance implies that factors associated with sex hormones may play a role in the development of glioma. The association between oral contraceptive (OC) use and glioma risk remains controversial.

**Method:**

In the Prostate, Lung, Colorectal, and Ovarian (PLCO) Cancer Screening Trial of 70,516 women in the USA, Cox proportional hazards regression analyses were adopted to calculate the crude and adjusted hazard ratios (HRs) and 95% confidence intervals (CIs). Additionally, a meta-analysis combining the PLCO findings with those of other prospective cohorts was performed.

**Results:**

During a mean follow-up of ~11.7 years, 110 of 70,516 women aged 50–78 years at baseline were diagnosed with glioma in PLCO studies. Compared with never users, an inverse association of borderline significance was found for OC users (HR 0.67, 95% CI 0.44–1.04, *P* = 0.074). Analyses assessing glioma risk according to the duration of OC use yielded no significant association. When PLCO was combined with four other prospective studies, there was an inverse association between OC use and glioma risk (HR 0.85, 95% CI 0.75–0.97, *I*^2^ = 0.0%). Further dose-response analysis showed a nonlinear, inverse relationship between OC use and glioma risk (*P* < 0.001).

**Conclusions:**

This study provided some evidence of a nonlinear, inverse association between OC use and glioma risk. Future larger studies are warranted to validate this finding.

## Introduction

Glioma is the most aggressive and most common brain malignancy and mainly comprises ependymoma, oligodendroglioma, astrocytoma, and oligoastrocytoma (1). The causative factors, apart from a strong link with ionizing radiation, remain largely unknown (2). Previous studies have suggested possible links between personal exposure and lifestyle and the development of glioma, but few well-established relationships have been identified (3).

Epidemiological studies have shown that the incidence of glioma, always exhibits sex disparity, with a 40%−50% lower incidence in women than in men (2). Experimental studies have shown the presence of progesterone and estrogen receptors in astrocytomas, and that metabolites of estradiol have a strong antiproliferative effect on glioma and induce an increase in apoptosis of human glioma cell lines (4, 5). These observations indicate that factors related to sex steroid hormones may play a role in the pathogenesis of glioma.

Oral contraception (OC) is a widely used, safe, and effective method to control birth, reduce menstrual symptoms, and regulate irregular or heavy menstruation (6, 7). Thus, additional estrogen and progestin exposures were present in women, and concerns about the risks or benefits of OC use were aroused. Researches on the effects of OC use on glioma risk have shown inconsistent findings (8–21). Notably, most previous studies were mainly retrospective case-control studies, and thus selection and recall bias may have distorted the findings. To strengthen the understanding of the possible effects of the OC on glioma risk, we assessed the association between OC and glioma risk in the Prostate, Lung, Colorectal, and Ovarian (PLCO) Cancer Screening Trial with a prospective design. We also performed a meta-analysis combining the PLCO with published prospective studies.

## Methods

### Data Sources

The PLCO study was a randomized controlled cancer screening trial. Detailed information about the rationale and design has been reported previously (22). A total of 154,887 subjects were recruited from 10 centers across the United States between 1993 and 2001. All eligible participants provided written informed consent and completed a self-reported baseline questionnaire, which mainly included information on demographics, body size (i.e., height, and weight), personal and family history of disease, smoking history, exogenous hormone use (in women), reproductive factors (in women), and prostate-related factors (in men). Ethical approval was obtained from the ethics committees of all PLCO cancer centers. The project number of the current study is PLCO-712.

### Study Population

**Figure 1:**
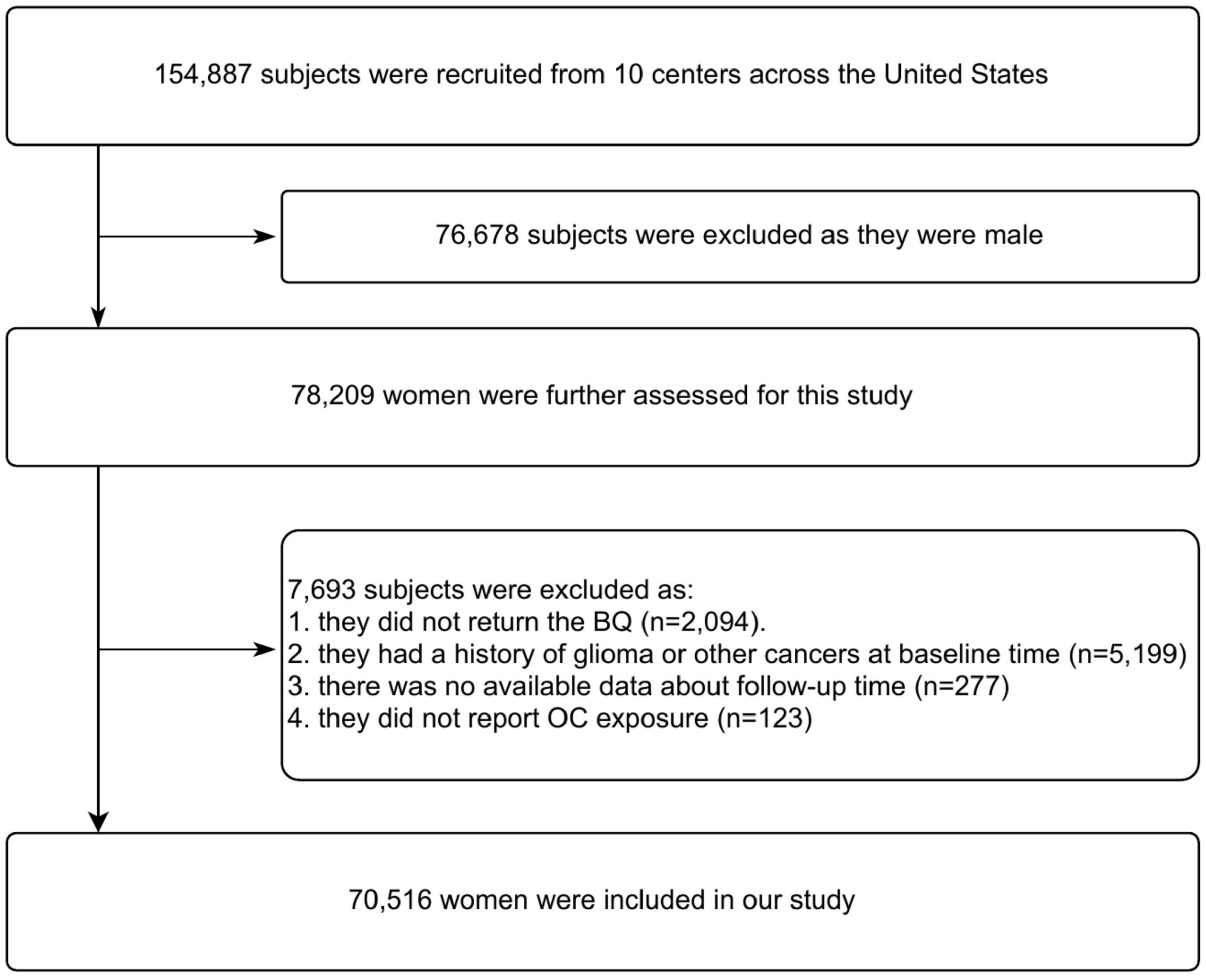
Flowchart of identifying subjects. BQ, baseline questionnaire; OC, oral contraception.

Figure 1 illustrates the flow chart of identifying subjects. At enrollment into the PLCO study, 78,209 of 154,887 subjects were women. According to our study design, we further excluded 7,693 subjects as they failed to return the baseline questionnaire (*n* = 2,094), had a history of glioma or other cancers (*n* = 5,199), and unavailable data about follow-up time (*n* = 277) or OC exposure (*n* = 123) was reported. Thus, data on 70,516 women aged 50–78 years at baseline were eligible for our analysis.

### Case Ascertainment

An annual follow-up questionnaire on cancer diagnosis was completed by all participants or their proxies. If someone was diagnosed with glioma, more information on diagnosis date, clinic or hospital center, and the doctor's contact information were identified. All glioma cases were verified through medical records. Follow-up was censored when participants died, participants were diagnosed with glioma, participants withdrew from the trial, or the trial ended (December 31, 2009), whichever occurred first.

### Exposure Assessment

Data about exogenous hormone use was retrospectively collected by a self-reported questionnaire at baseline. All female subjects were asked whether they had ever used OC and hormone replacement therapy (HRT), age at first use, and years of use in the baseline questionnaire. Information on the type of hormone was not collected. We performed an analysis for ever use and duration of ever use (<5 vs. 6–9 vs. ≥10 years) compared with never use of OC.

### Statistical Analyses

Differences in baseline characteristics between OC users and nonusers were analyzed using χ^2^ tests for categorical variables. Cox proportional hazards regression analyses were adopted to calculate the crude and adjusted hazard ratios (HRs) and 95% confidence intervals (CIs). The following covariates, including age (smooth), race (White, non-Hispanic vs. other/unknown), marital status (married or living as married vs. never married), education (up to high school vs. some college or post-high school training vs. at least college graduate), smoking status (never vs. ever smoked), HRT (ever vs. never use), and body mass index (BMI; <25 kg/m^2^ vs. ≥25 kg/m^2^) were assessed in the adjusted model. Subgroup analysis was performed according to the BMI and HRT use. All analyses were performed by R software (version 3.4.3; http://www.R-project.org) and Empower (version 2.0; X&Y Solutions, Inc. Boston, MA, USA).

### Meta-Analysis

To retrieve studies on the relationship between OC use and glioma risk, we performed a literature search of PubMed and EMBASE databases (from their inception to Jan. 22, 2022). The detailed search string was: [hormone (Title/Abstract) OR contraceptive (Title/Abstract)] AND [“brain tumor” (Title/Abstract) OR “brain cancer” (Title/Abstract) OR “brain neoplasms” OR glioma (Title/Abstract)]. Other than limiting to human studies, no other limits were imposed. We also reviewed the reference lists of potential studies, reviews, and meta-analyses associated with this topic. Prospective cohort studies providing adjusted HRs with 95% CIs (or other data about exposure distribution to calculate them) were considered to be eligible for meta-analysis. Two reviewers independently and carefully extracted the following data: first author, publication year, study country, study design, study period, sample size, exposure variables, the most fully adjusted risk estimates with 95% CIs, and adjustment factors. Any disagreements were settled by discussion. The quality assessment of all eligible studies was performed through the Newcastle–Ottawa Scale (http://www.ohri.ca/programs/clinical_epidemiology/oxford.asp, accessed on 13 June 2022). The scoring system includes selection (a maximum of four stars), comparability (a maximum of two stars), and outcome (a maximum of three stars). In this study, we also made some modifications to this guideline tool, including the following aspects: (1) a maximum of one star was awarded to a study in which adjusted risk estimates were reported, as confounding factors are one of the most biggest concerns in observational studies; and (2) a study with follow-up (greater than the median or mean follow-up of 5 years) was awarded one star.

We combined the risk estimates with corresponding 95% CIs with a random-effects model by DerSimonian and Laird (23), when the value of *I*^2^ statistic was >50% (24); otherwise, a fixed-effects model was adopted. Potential publication bias was evaluated by Begg's funnel plots and Egger's test, while publication bias was not evaluated when there were <10 included studies. We also assessed the influence of single studies by excluding one study at a time. As the most frequent definition of OC exposure was users vs. nonusers, we first performed an analysis of comparison of OC users vs. nonusers. When some studies only provided risk estimates for current use and past use vs. no use, we finished data conversion representing ever use vs. no use as described previously (25, 26). Briefly, we pooled the risk estimates using a fixed-effects model before combining with other studies. To determine the causality of an association, we tried to identify a dose-response relationship between OC use and glioma with a one-stage robust error meta-regression model proposed by Doi (27). This method treats each study as a cluster and weighs the effects of each study by its inverse variance, while employs the robust-variance to address the potential correlation of the within-study effects. Also, it requires at least two categories for each included study. When a specific exposure level was reported as a range, the median or mean was assigned. When the highest or lowest exposure level was open-ended, its width matching the interval of the adjacent category was assumed. All analyses for meta-analysis were performed using STATA software (version 15.0, STATA Corp., College Station, TX, USA).

## Results

### PLCO Trial

A total of 70,516 women aged 50–78 years at baseline were eligible for our analysis. Of these, 110 women were diagnosed with incident glioma during a mean follow-up of ~11.7 years. Baseline cohort characteristics are shown in Table 1. Most participants were younger than 65 years and were White, non-Hispanic. Women with HRT use were more likely to be OC users.

**Table 1:** Baseline participant characteristics according to OC use in the PLCO trail.

Characteristic^*^	**OC use**		***P*** **-value**
	**Never use (*n* = 32,206)**	**Ever user (*n* = 38,310)**	
**Age (years)**, ***n*** **(%)**			
<65	15,493 (48.11%)	30,156 (78.72%)	<0.001
≥65	16,713 (51.89%)	8,154 (21.28%)	
**Arm**, ***n*** **(%)**			
Intervention	16,237 (50.42%)	19,092 (49.84%)	0.125
Control	15,969 (49.58%)	19,218 (50.16%)	
**Race**, ***n*** **(%)**			
White, non-Hispanic	28,068 (87.15%)	34,325 (89.60%)	<0.001
Others/unknown	4,138 (12.85%)	3,985 (10.40%)	
**Education**, ***n*** **(%)**			
Up to high school	12,522 (38.97%)	11,584 (30.29%)	<0.001
Some college or post high school training	11,004 (34.25%)	14,332 (37.47%)	
At least college graduate	8,607 (26.79%)	12,329 (32.24%)	
**Marital status**, ***n*** **(%)**			
Married or living as married	30,511 (94.90%)	37,552 (98.15%)	<0.001
Never married	1,641 (5.10%)	706 (1.85%)	
**Smoking status**, ***n*** **(%)**			
Never	19,643 (61.00%)	19,879 (51.89%)	<0.001
Ever	12,558 (39.00%)	18,430 (48.11%)	
**BMI (kg/m**^**2**^**)**, ***n*** **(%)**			
<25	12,855 (40.55%)	15,769 (41.64%)	0.004
≥25	18,845 (59.45%)	22,099 (58.36%)	
**HRT**, ***n*** **(%)**			
Never	13,170 (41.14%)	9,865 (25.85%)	<0.001
Ever	18,841 (58.86%)	28,295 (74.15%)	

*OC, oral contraception; PLCO, prostate, lung, colorectal and ovarian; BMI, body mass index; HRT, hormone replacement therapy; n, number*.

* *There were 138, 106, 948, 6, and 345 subjects with missing data for education, marital status, BMI, smoking status, and HRT use, respectively*.

Prior to adjustment for any potential confounders, ever OC use was inversely associated with risk of glioma (HR 0.63, 95% CI 0.43–0.94), and some borderline significant results were observed for the duration of OC use (Table 2). Similarly, an inverse association of borderline significance was found for ever users in the multivariable analysis. Further analyses assessing glioma risk according to the duration of OC use yielded no significant association between OC and glioma risk (Table 2).

**Table 2:** OC use and glioma risk in the PLCO trail.

**Exposure**	**Cohort (*n*)**	**Cases (*n*)**	**Non-adjusted HR (95% CI) and *P*-value**	**Adjusted HR (95% CI) and *P*-value**
**OC use**				
Never	32,206	58	1.0	1.0
Ever	38,310	42	0.63 (0.43, 0.94) 0.025	0.67 (0.44, 1.04) 0.074
**Years of OC**				
Never	32,206	58	1.0	1.0
≤5	23,087	25	0.63 (0.39, 1.00) 0.051	0.66 (0.40, 1.09) 0.104
6–9	6,307	9	0.82 (0.41, 1.67) 0.591	0.91 (0.44, 1.88) 0.798
≥10	8,841	8	0.52 (0.25, 1.10) 0.086	0.56 (0.26, 1.19) 0.131

*PLCO, prostate, lung, colorectal and ovarian; HR, HR, hazard ratio; 95% CI, 95% confidence interval; OC, oral contraception; HRT, hormone replacement therapy; BMI, body mass index; n, number*.

*Adjusted for age (smooth), race, marital status, education, BMI, HRT, and smoking*.

Table 3 shows the subgroup results according to BMI. The association between OC use and glioma risk was more pronounced in women with BMI ≥25 kg/m^2^ (adjusted HR 0.46, 95% CI 0.25–0.83), but not with BMI <25 kg/m^2^ (adjusted HR 1.11, 95% CI 0.58–2.15). When assessing the duration of OC use, no clear dose-response relationship was identified. Another subgroup analysis by HRT use showed no significant difference between ever users and never users of HRT, although none of the risk estimates was significant (Table 4).

**Table 3:** OC use and glioma risk according to BMI in the PLCO trail.

**Exposure**	**BMI <25 kg/m** ^**2**^		**BMI ≥25 kg/m** ^**2**^	
	**Crude HR (95% CI) and *P*-value**	**Adjusted HR (95% CI) and *P*-value**	**Crude HR (95% CI) and *P*-value**	**Adjusted HR (95% CI) and *P*-value**
**OC use**				
Never use	1.0	1.0	1.0	1.0
Ever use	0.98 (0.53, 1.82) 0.954	1.11 (0.58, 2.15) 0.746	0.43 (0.25, 0.75) 0.003	0.46 (0.25, 0.83) 0.010
**Years of OC use**				
Never use	1.0	1.0	1.0	1.0
≤5	0.84 (0.40, 1.76) 0.636	0.97 (0.44, 2.10) 0.929	0.48 (0.26, 0.91) 0.023	0.51 (0.26, 0.99) 0.048
6–9	1.62 (0.65, 4.06) 0.304	1.87 (0.72, 4.84) 0.196	0.42 (0.13, 1.35) 0.144	0.43 (0.13, 1.44) 0.173
≥10	0.91 (0.34, 2.45) 0.855	0.99 (0.36, 2.73) 0.986	0.31 (0.10, 1.00) 0.050	0.32 (0.10, 1.07) 0.065

*PLCO, prostate, lung, colorectal and ovarian; HR, hazard ratio; 95% CI, 95% confidence interval; OC, oral contraception; HRT, hormone replacement therapy; BMI, body mass index*.

*Adjusted for age (smooth), race, marital status, education, HRT, and smoking*.

**Table 4:** OC use and glioma risk according to HRT in the PLCO trail.

**Exposure**	**Nonusers**		**Ever users**	
	**Crude HR (95% CI) and *P*-value**	**Adjusted HR (95% CI) and *P*-value**	**Crude HR (95% CI) and *P*-value**	**Adjusted HR (95% CI) and *P*-value**
**OC use**				
Never use	1.0	1.0	1.0	1.0
Ever use	0.49 (0.22, 1.11) 0.087	0.54 (0.23, 1.26) 0.151	0.66 (0.42, 1.06) 0.089	0.73 (0.44, 1.22) 0.228
**Years of OC use**				
Never use	1.0	1.0	1.0	1.0
≤5	0.39 (0.13, 1.13) 0.083	0.43 (0.14, 1.27) 0.127	0.69 (0.40, 1.19) 0.184	0.75 (0.42, 1.34) 0.340
6–9	0.39 (0.05, 2.90) 0.358	0.42 (0.06, 3.21) 0.406	0.94 (0.43, 2.01) 0.864	1.08 (0.49, 2.38) 0.857
≥10	0.89 (0.27, 2.98) 0.851	0.96 (0.28, 3.31) 0.945	0.41 (0.16, 1.04) 0.061	0.46 (0.18, 1.19) 0.107

*PLCO, prostate, lung, colorectal and ovarian; HR, hazard ratio; 95% CI, 95% confidence interval; OC, oral contraception; HRT, hormone replacement therapy; BMI, body mass index*.

*Adjusted for age (smooth), race, marital status, education, BMI, and smoking*.

### Meta-Analysis

**Figure 2:**
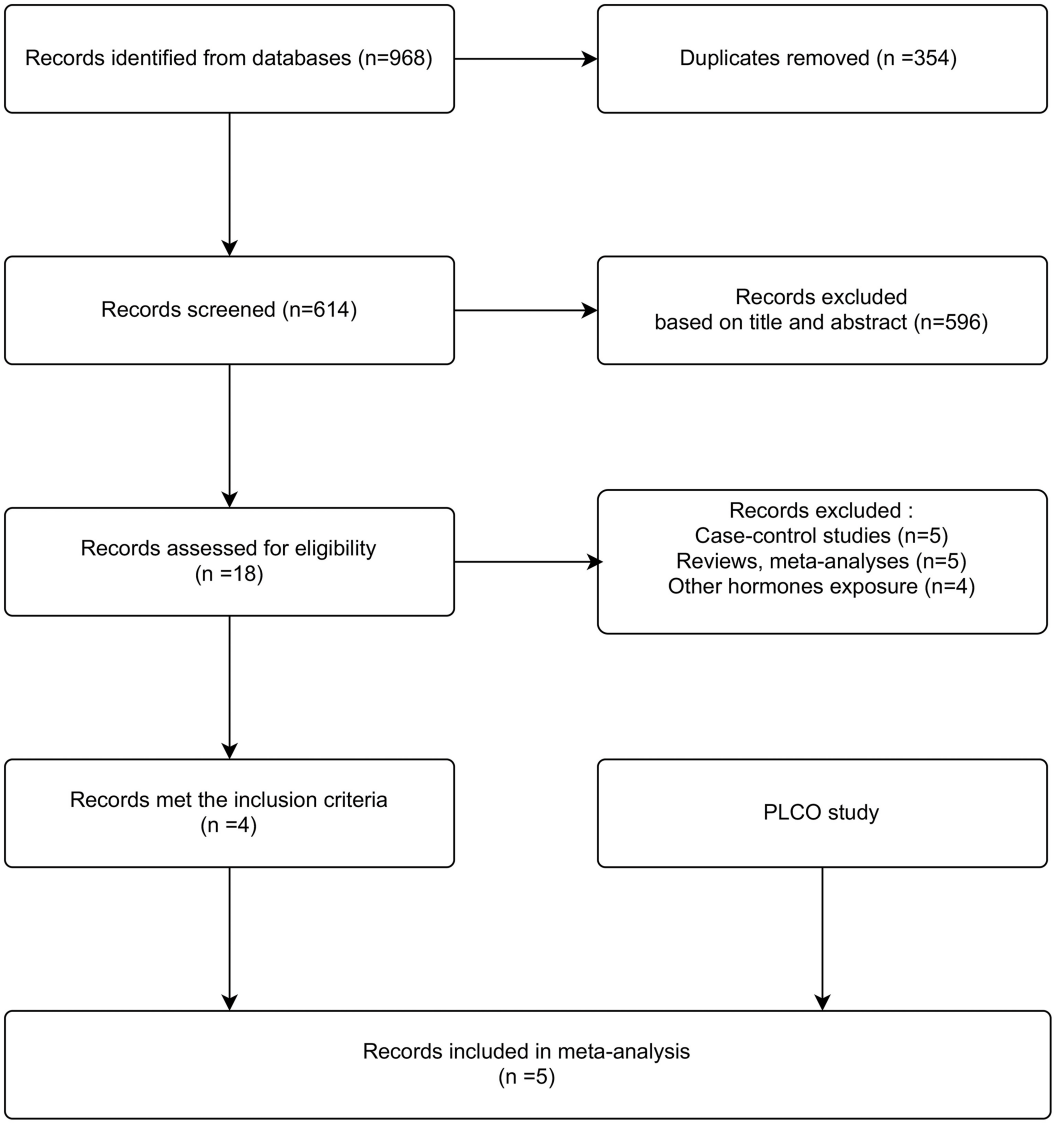
Flowchart for the study selection process.

The process of study selection is shown in Figure 2. The initial literature search of PubMed (*n* = 417) and EMBASE (*n* = 551) databases identified 968 records. After careful review, four studies met the inclusion criteria (18–21), and including PLCO, five were accepted for analysis. The basic characteristics of included studies were shown in Supplementary Table 1. These studies were conducted in the USA, Sweden, Denmark, Canada, the UK, Germany, Netherlands, Greece, Italy, Norway, and Spain. At baseline, women aged 20–80 years were recruited. More than 90% women were White, non-Hispanic in the NIH-AARP Diet and Health Study (21), while no detailed proportion was reported in other studies (18–20). The mean follow-up time ranged from 6.2 to 11.6 years. A total of 1,263 gliomas were diagnosed. Glioma cases were ascertained by cancer registrations, health insurance records, national mortality databases, or pathology registries. OC exposure was assessed by a self-administered/reported questionnaire. All included studies reported nonusers of OC as the reference category. The highest exposure category ranged from more than 3 to 15 years. All studies were awarded seven stars, indicating that the included studies were of high quality (Supplementary Table 2).

**Figure 3:**
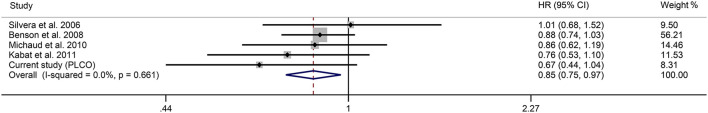
Forest plots for the relationship between OC use and glioma risk. OC, oral contraception.

**Figure 4:**
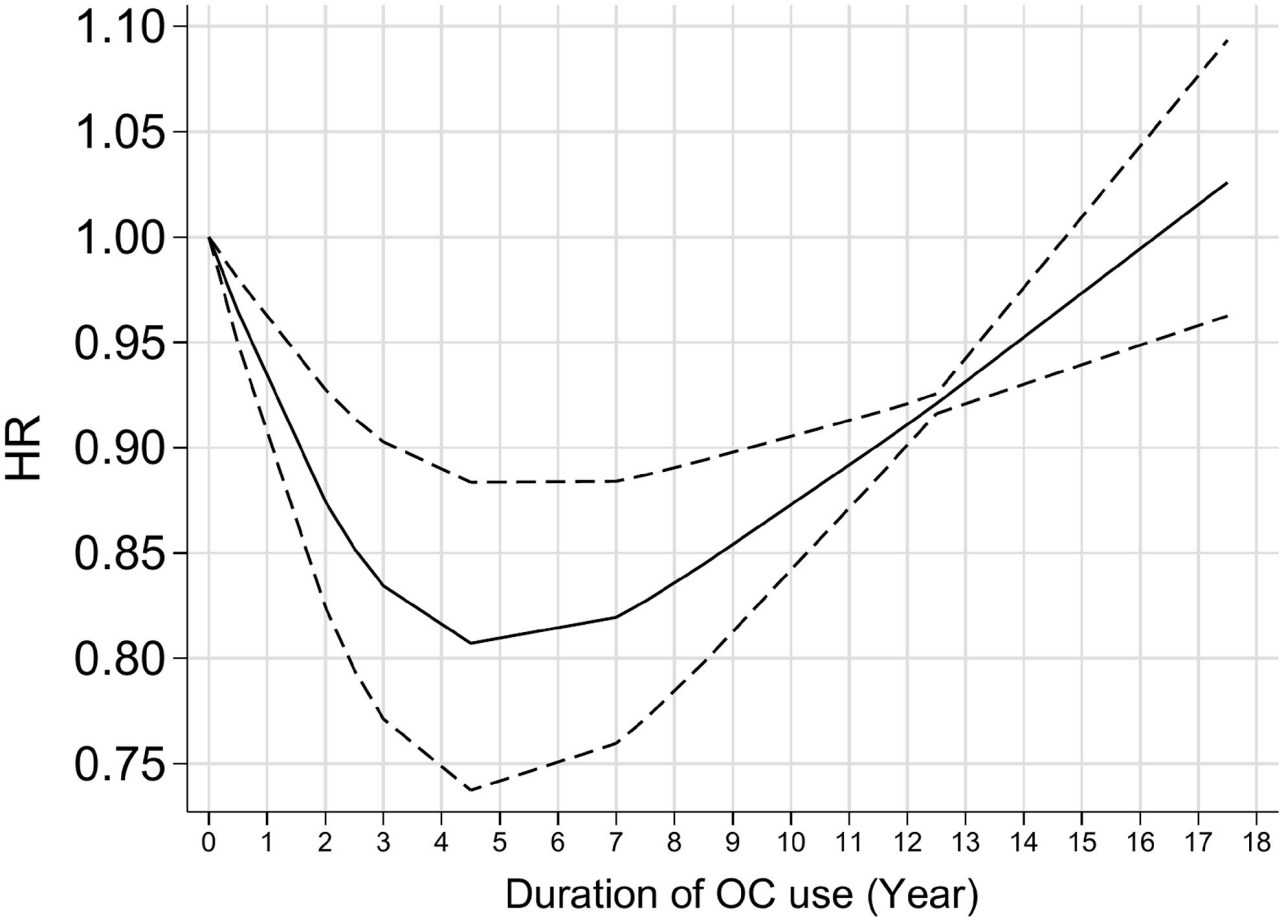
Dose-response relationships between OC use and glioma risk. OC, oral contraception.

Figure 3 shows the forest plot for the effect of OC use on glioma risk. The pooled results gave a decreased risk of 0.85 with OC users compared with nonusers (95% CI 0.75–0.97, *I*^2^ = 0.0%). Further analysis showed that the overall results were not influenced by a single study, indicating that the results were stable (Supplementary Table 3). Dose-response analysis showed a nearly “U” shaped association between glioma risk and OC use (Figure 4, *P* for nonlinearity <0.001). Specifically, the strongest inverse relationship was found in women who took the OC pills for 4–5 years. There was no further reduction in glioma risk with increasing duration of OC use over 4–5 years.

## Discussion

In the PLCO study, we found that the use of OC was not significantly associated with glioma risk. In the subsequent meta-analysis that incorporated our study, we found there was an inverse association between glioma risk and OC use. Dose-response analysis showed a nearly “U” shaped association between glioma risk and OC use.

The relationship between OC use and glioma risk has been reported with inconsistent results (8–21). In line with PLCO, nearly all previous studies showed a weak inverse association or no significant association between OC use and glioma risk, except one reporting an increased risk of glioma with OC use (17). To the best of our knowledge, we first reported that BMI was as an effect modifier on the association between glioma risk and OC use. The association between OC use and glioma risk was more pronounced in women with BMI ≥25 kg/m^2^. However, there was a positive association between glioma risk and OC use, albeit insignificant. One potential explanation is that this discrepancy was associated with additional estrogen exposure in overweight women. Excess adipose tissue can cause increased production of estrogen through higher conversion rates of androgens due to increased aromatase activity, which results in higher aromatization of androstenedione (28–31). Another explanation is that the subgroup results were chance findings.

Other than our study, only two case-control studies examined the potential interaction between postmenopausal hormone use and OC use (13, 16). In the large San Francisco Bay Area Adult Glioma Study with 619 cases and 650 controls, Felini et al. found that exogenous hormone use was inversely associated with glioma risk, but there was no evidence of antagonism or synergy effect between previous OC use and HRT and glioma (13). We also found that HRT did not modify the association between glioma risk and OC use. On the contrary, another study showed that users of both OC and HRT were less likely to have glioma (16), compared to those with neither OC nor HRT use (16).

Currently, the components of OC tablets differ markedly from those used previously. When the OCs entered the market in the 1960s, estrogen concentration in earlier generations was >100 μg (32). Today, the OCs contain lower levels of estrogen (<35 μg) and progestin (32). There is some evidence that the expression of estrogen and progesterone receptors varies in glioma, and for example, higher-grade glioma is associated with lower expression of estrogen receptors and higher expression of progesterone receptors (4). Also *in vitro* studies, a dual role of progesterone in glioblastoma cells has been suggested: physiological concentrations increase cell proliferation, invasion, and migration, while higher doses beyond the physiological level inhibit cell proliferation and promote cell death (33). To our knowledge, only one case-control study based on the nationwide prescription registry assessed the effect of hormonal contraceptive components on glioma risk (17). The positive association between glioma risk and long-term use of hormonal contraceptive was most pronounced for progestogen-only therapy (17). Hence, women with different hormone exposure may also bear a different risk of glioma and more studies are needed to clarify this issue.

Another concern is the association between the state of OC use and glioma risk. Three studies provided limited data for current use vs. past/former use of OC. One prospective European investigation with 276,212 individuals reported no significant difference between past and current OC users (20). Another case-control study reported no association among past users, but a significant inverse association among current users (13). Additionally, a more obvious dose–response trend of decreasing risk with a longer duration of use was identified among current users (13). On the contrary, the national prescription registry case-control study showed that the increased risk was higher for current/recent use rather than for past users (17). These findings indicate that the risk may not change over time after OC discontinuation. Therefore, caution is warranted regarding the findings and ongoing discussion about the duration of OC use and glioma risk is needed.

Several meta-analyses on the association between OC and glioma risk have been published (34–37). Similarly, these results were mainly based on retrospective studies and showed a preventive effect of OC on glioma risk. The latest meta-analysis of eight retrospective case-control and four prospective cohort studies published in 2021 showed a nonlinear correlation between glioma risk and duration of OCs use (37). Specifically, significant benefits of a decreased risk of glioma were observed in women with longer-term OC use, with a “time window” of over 7.5 years (37). In the current study, we pooled the PLCO and other prospective cohort studies and found a nearly “U” shaped association between OC and glioma risk. The strongest inverse association was found in women who took the OC pills for 4–5 years and when using OC pills more than 4–5 years, the risk of glioma does not decrease further.

Advantages of the PLCO study included its prospective design, longer duration of follow-up, and consideration of some essential confounders. Several limitations should be mentioned as well. First, there were small numbers of glioma cases in the PLCO trial and meta-analysis, which may have led to chance results for OC use. Concerning that little is known about the etiology, this study provided some insight into the role of female hormone on glioma risk. Second, all self-reported exposures at baseline always have bias concerns; thus, measurement error or misclassification may be inevitable. Third, uncontrolled confounders may belie the true association between OC use and glioma risk. Finally, the potential publication bias was not addressed, as few studies were included in this meta-analysis.

In conclusion, our study provided some evidence of an inverse association between OC use and glioma risk. Additional prospective studies with a large sample size are warranted to confirm our findings, especially addressing the components of OC and the dose-response relationship.

## Data Availability Statement

The data of the current study are available from the NIH PLCO study group subject to restrictions, as the data were used under license for the current study. Requests to access the datasets should be directed at: https://cdas.cancer.gov/plco/.

## Ethics Statement

Ethical approval was obtained from the Ethics Committees of all PLCO cancer centers. The patients/participants provided their written informed consent to participate in PLCO study at study entry.

## Author Contributions

CS, HT, and XW contributed to conception and design of the study. CS, HT, XW, and PW performed data cleaning and statistical analysis. CS, HT, XW, PW, NW, and JH wrote the first draft of the manuscript. PW and NW funding acquisition. All authors contributed to manuscript revision, read, and approved the submitted version.

## Conflict of Interest

The authors declare that the research was conducted in the absence of any commercial or financial relationships that could be construed as a potential conflict of interest.

## Publisher's Note

All claims expressed in this article are solely those of the authors and do not necessarily represent those of their affiliated organizations, or those of the publisher, the editors and the reviewers. Any product that may be evaluated in this article, or claim that may be made by its manufacturer, is not guaranteed or endorsed by the publisher.
