# Supplementary material for: Oral Contraceptive and Glioma Risk: A Prospective Cohort Study and Meta-Analysis
Source: Front Public Health. 2022 Jul 14;10:878233. doi: 10.3389/fpubh.2022.878233 (PMC9330220; doi:10.3389/fpubh.2022.878233)
Supplement: Supplementary file 1 [file Data_Sheet_1.docx]

**Supplementary Material**

Supplementary Table 1 Basic characteristics of four included studies.

| Reference | Country | Follow-up (years) | Age (years) | Sample size | Cases ascertainment | Exposure assessment | Adjusted factors |
| --- | --- | --- | --- | --- | --- | --- | --- |
| Silvera et al. 2006 [18] | Canada | 16.4 | 40-59 | 120/89,835 | National cancer and mortality database | Self-reported questionnaire | Age, study center, randomization group, parity, age at menarche, menopausal status. |
| Benson et al. 2008 [19] | the UK | 6.2 | 50-65 | 646/1,249,670 | Cancer registrations | Self-reported questionnaire | Age, region, height, body mass index, socioeconomic status, smoking status, alcohol intake, strenuous exercise, age at first birth, and parity. |
| Kabat et al. 2010 [20] | USA | 7.5 | 50-71 | 174/ 225,355 | Cancer registries | Self-reported questionnaire | Age, race, age at menarche, parous/nulliparous, age at menopause, history of hysterectomy, and smoking status. |
| Michaud et al. 2010 [21] | European | 8.4 | 20-83 | 193/276,212 | Histologically confirmed | Self-reported questionnaire | Age, education, body mass index, and menopausal status smoking status, education, body mass index and menopausal status |

Supplementary Table 2 Methodological quality of included studies based on the Newcastle–Ottawa Scale

| Reference | Selection | Comparability | Outcome | Total |
| --- | --- | --- | --- | --- |
| Silvera et al. 2006 [18] | *** | * | *** | 7* |
| Benson et al. 2008 [19] | *** | * | *** | 7* |
| Kabat et al. 2010 [20] | *** | * | *** | 7* |
| Michaud et al. 2010 [21] | *** | * | *** | 7* |
| PLCO study | *** | * | *** | 7* |

Supplementary Table 3 Result of assessing the robustness.

| Study omitted | HR and 95% CI | I^2^ |
| --- | --- | --- |
| Silvera et al. 2006 | 0.84(0.74-0.96) | 0.0% |
| Benson et al. 2008 | 0.82(0.68-0.99) | 0.0% |
| Michaud et al. 2010 | 0.85(0.75-0.98) | 0.0% |
| Kabat et al. 2011 | 0.87(0.76-0.99) | 0.0% |
| Current study (PLCO) | 0.87(0.77-0.99) | 0.0% |
